# Supplementary material for: Autonomous self-healing organic crystals for nonlinear optics
Source: Nat Commun. 2023 Oct 18;14:6589. doi: 10.1038/s41467-023-42131-7 (PMC10584936; doi:10.1038/s41467-023-42131-7)
Supplement: Supplementary file 1 — Supplementary Information [file 41467_2023_42131_MOESM1_ESM.pdf]

Supplementary Information

Saikat Mondal[1], Pratap Tanari[1], Samrat Roy[2], Surojit Bhunia[1], Rituparno Chowdhury[1], Arun K. Pal[3], Ayan Datta[3], Bipul Pal[2]* and C. Malla Reddy[1]*

[1]Department of Chemical Sciences, Indian Institute of Science Education and Research Kolkata, Nadia 741246, West Bengal, India.

[2]Department of Physical Sciences, Indian Institute of Science Education and Research Kolkata, Nadia 741246, West Bengal, India.

[3]School of Chemical Sciences, Indian Association for the Cultivation of Science, Kolkata 700032, West Bengal, India.

E-mail: bipul@iiserkol.ac.in; cmallareddy@gmail.com

List of Supplementary informations

Sl. No.	Contents	Page No.
1.	**Supplementary Fig. 1: Thermal analysis of *1***	2
2.	**Supplementary Fig. 2: SEM images of different healed crystals**	3
3.	**Supplementary Fig. 3: SEM images of different imperfectly healed crystals**	4
4	**Supplementary Fig. 4: KPFM studies on a freshly created surface (001) face of a crystal of *1***	5
5.	**Supplementary Fig. 5, 6: Healing time calculation**	6-7
6.	**Supplementary Fig. 7, 8: Calculation of actuation parameters**	8-9
7.	**Supplementary Fig. 9: Response time comparision**	10
8.	**Supplementary Fig. 10: Examining self-healing efficiency using crystallographic diffraction experiments**	11
9.	**Supplementary Fig. 11, 12, 13, 14: SHG setup, SHG analysis and comparisons**	12-15
10.	**Supplementary Fig. 15: Videograbs of healed and imperfectly healed crystals and their corresponding SPM images**	16
11.	**Supplementary Fig. 16: PXRD**	17
12.	**Supplementary Fig. 17: Histogram plots of elastic modulus and hardness**	18
13.	**Supplementary Table 1. Quantified loads for multiple healing cycles and fracture for different crystals of *1***	19
14.	**Supplementary Table 2. Ranges of materials properties and certain accessible performance attributes**	20
15.	**Supplementary Table 3, 4, 5, 6: Crystallographic information**	21-24
16.	**Supplementary Table 7, 8: Statistical calculation for the actuation motions**	25-26
17.	**Supplementary References**	26

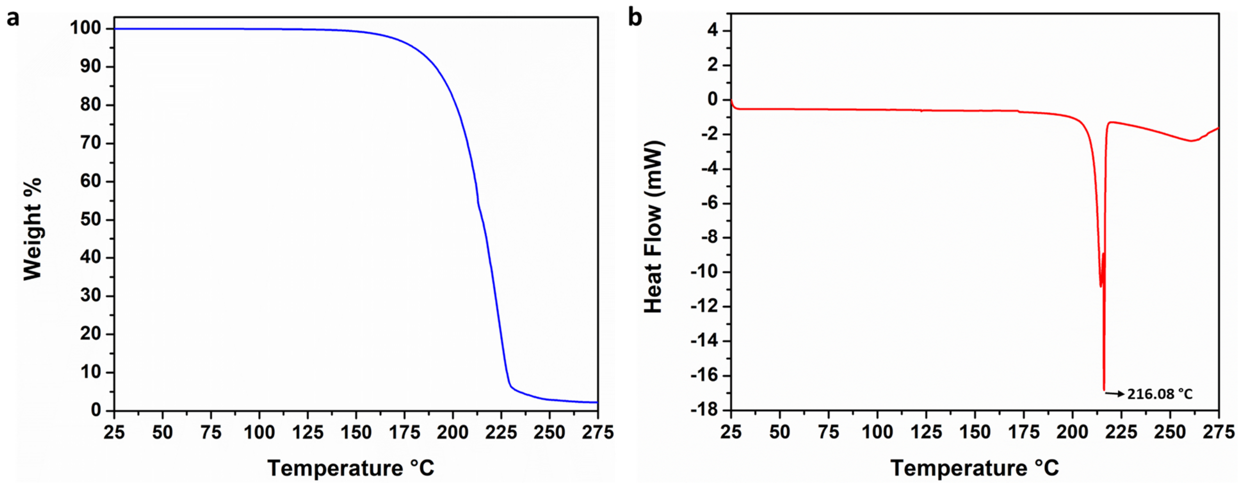

Supplementary Fig. 1: Thermal analysis of ***1*****. a** TGA plot of ***1*** (blue) and **b** corresponding endotherms in DSC (red).

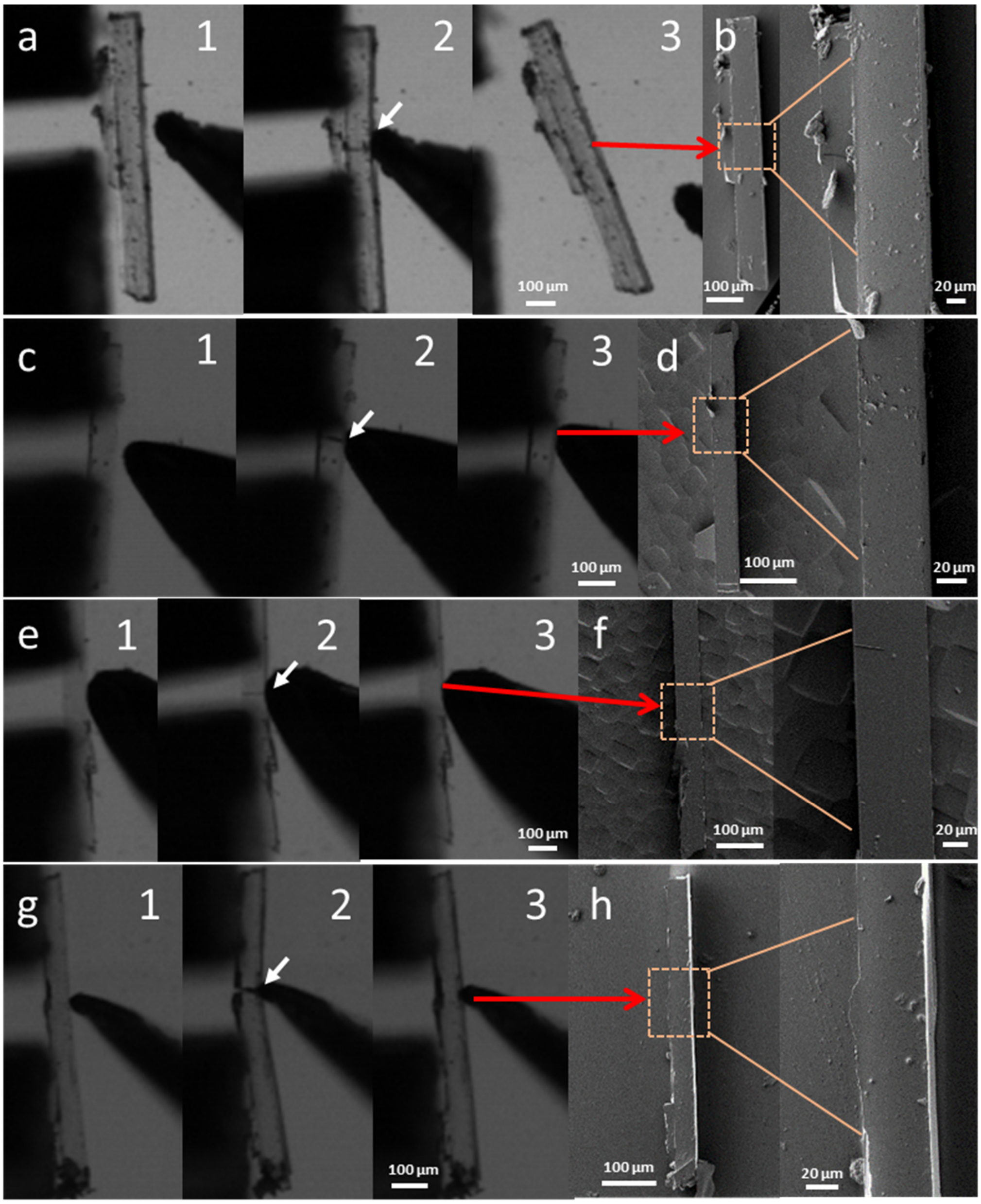

Supplementary Fig. 2: SEM images of different healed crystals. a, **c**, **e**, **g** are the videograbs of different crystals, which eventually healed during mechanical manipulation, **b**, **d**, **f**, **h** are their corresponding SEM images.

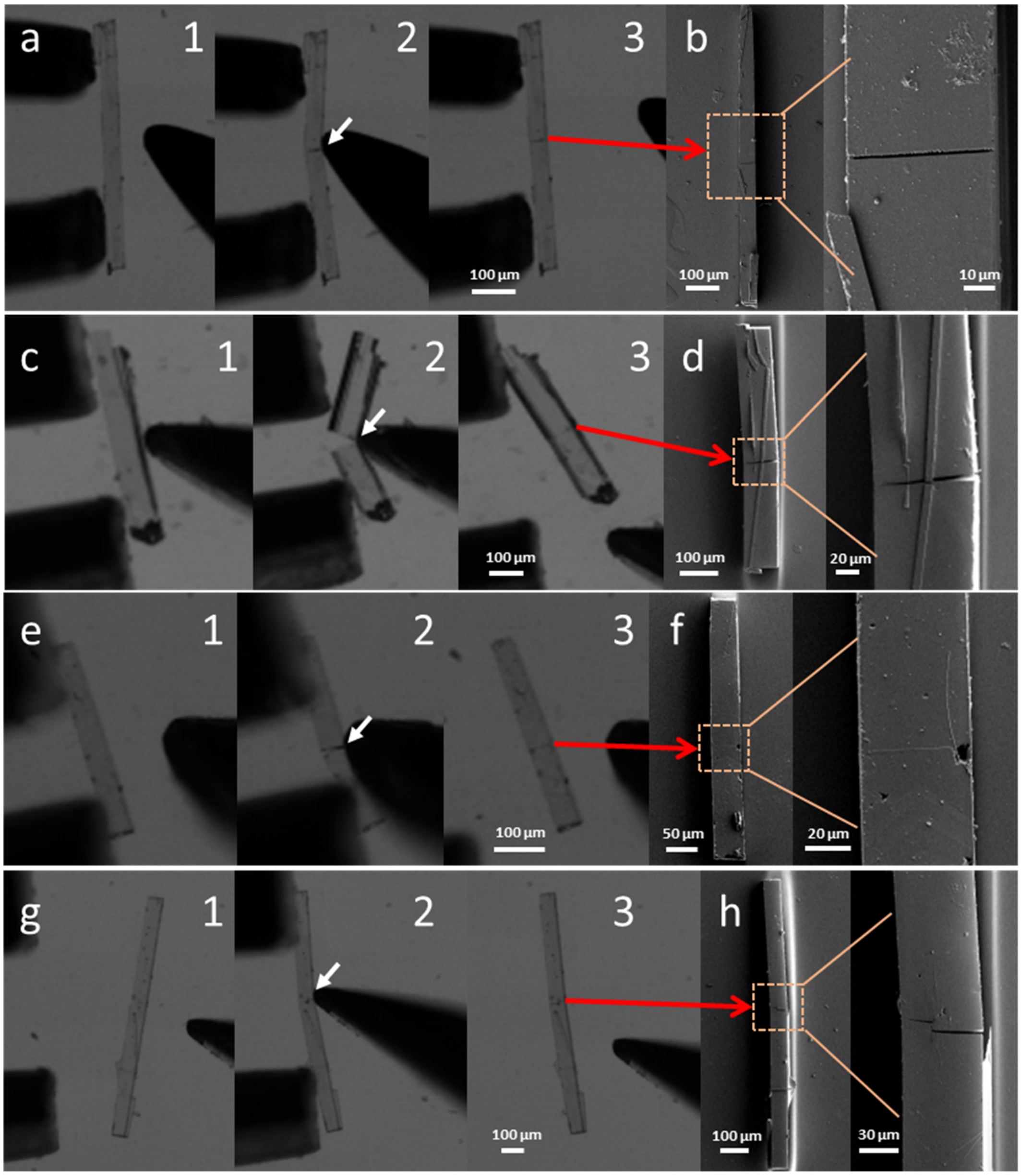

Supplementary Fig. 3: SEM images of different imperfectly healed crystals. a, **c**, **e**, **g** are the videograbs of different crystals, which failed to heal after excessive mechanical loading during the manipulation, **b**, **d**, **f**, **h** are their corresponding SEM images.

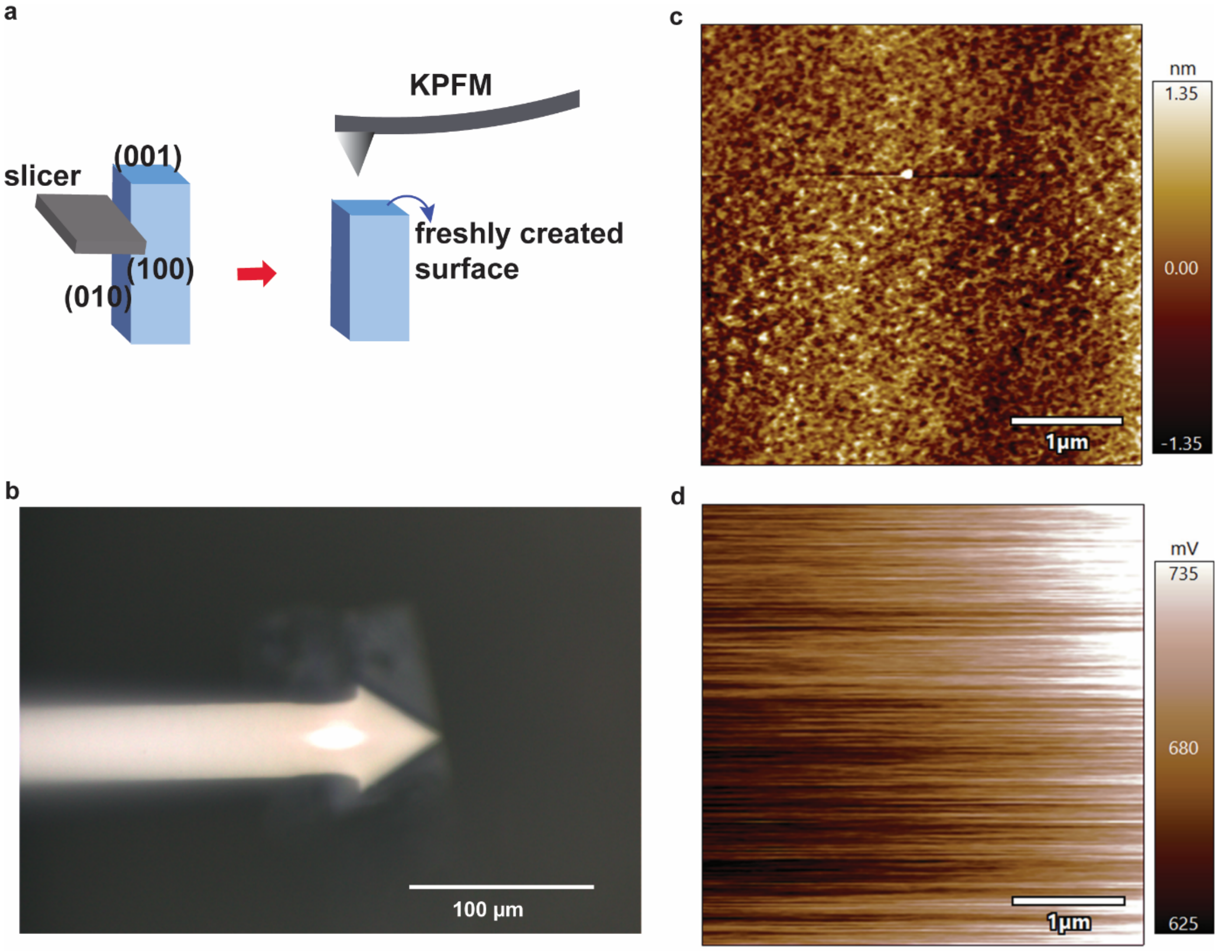

Supplementary Fig. 4: KPFM studies on a freshly created surface (001) of a crystal of *1*. (a) Schematic of the preparation of sample for KPFM measurements. Measurement is performed on a freshly created surface of (001) by mechanically fracturing the crystal of ***1***, which was mounted vertically using silver paste on a FTO coated glass. (b) Optical image of the AFM cantilever (focused) and freshly created surface of (001) of a crystal of ***1*** (in out of focus), which was taken prior to performing the experiment. (c) Topography image and (d) surface potential (average surface potential is around + 0.68 V) image of a scan area of 4 μm x 4 μm.

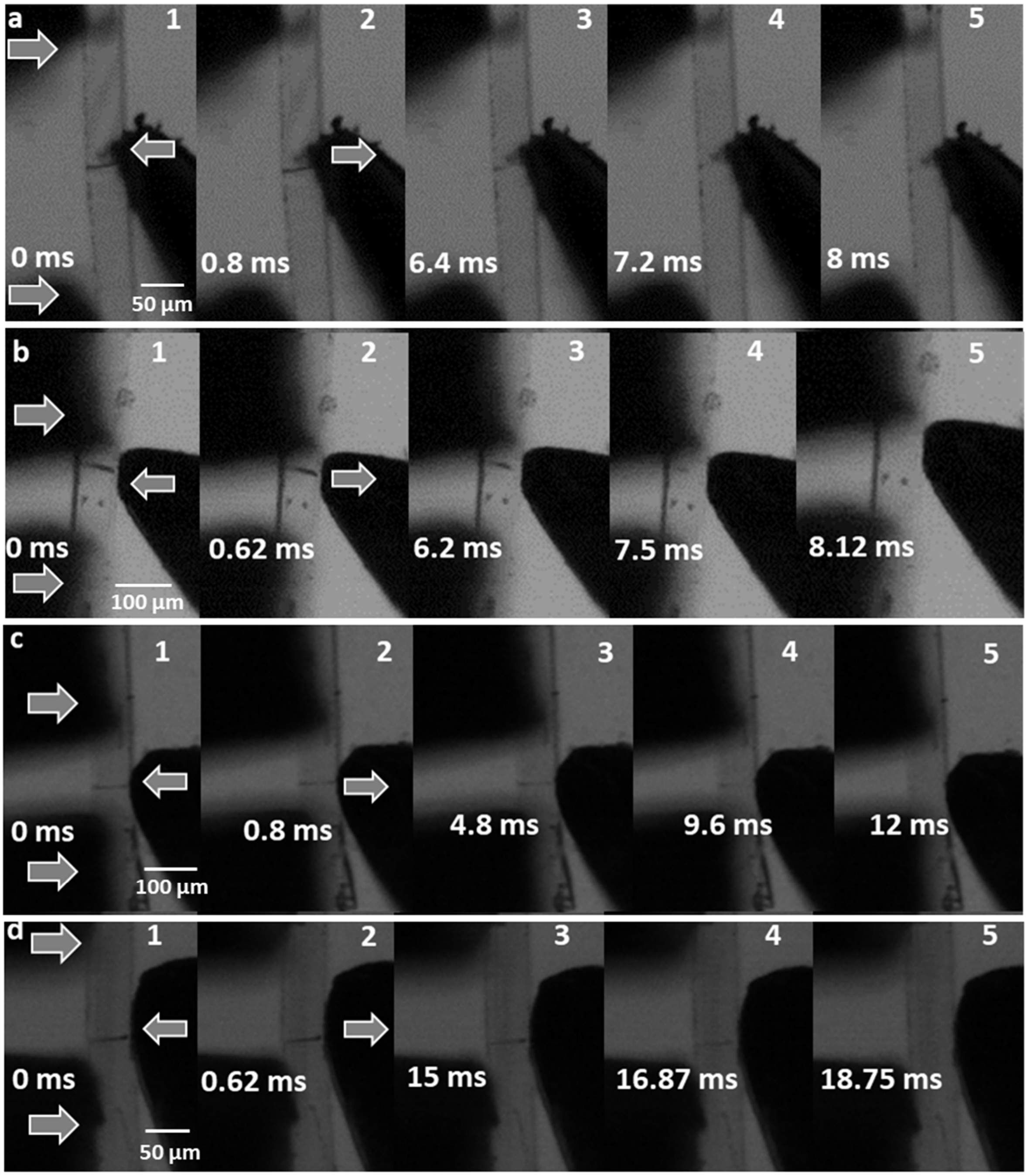

Supplementary Fig. 5: The videograbs of healing events of multiple single crystals {**a**, **b**, **c** and **d**} captured using a slow motion digital camera to calculate healing time.

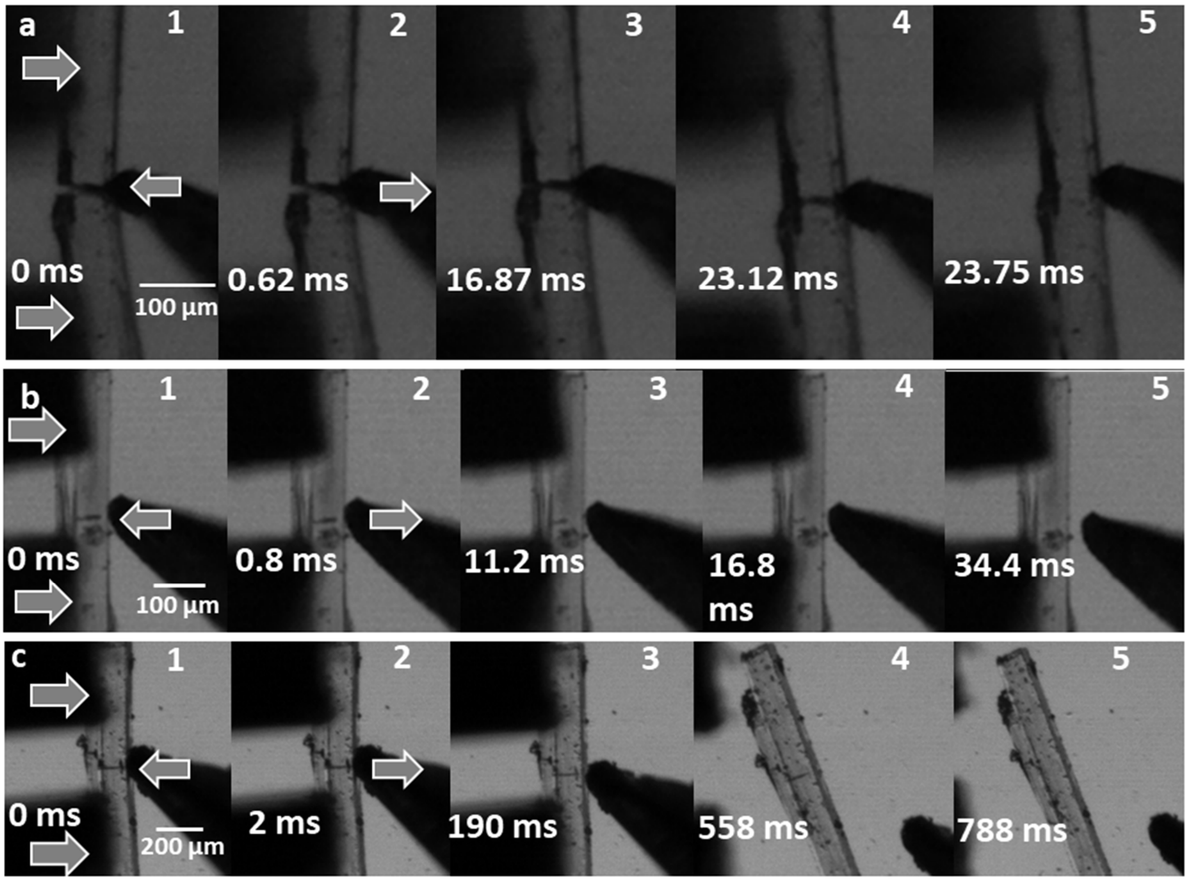

Supplementary Fig. 6: The videograbs of healing events of multiple single crystals {**a**, **b** and **c**} captured using a slow motion digital camera to calculate healing time.

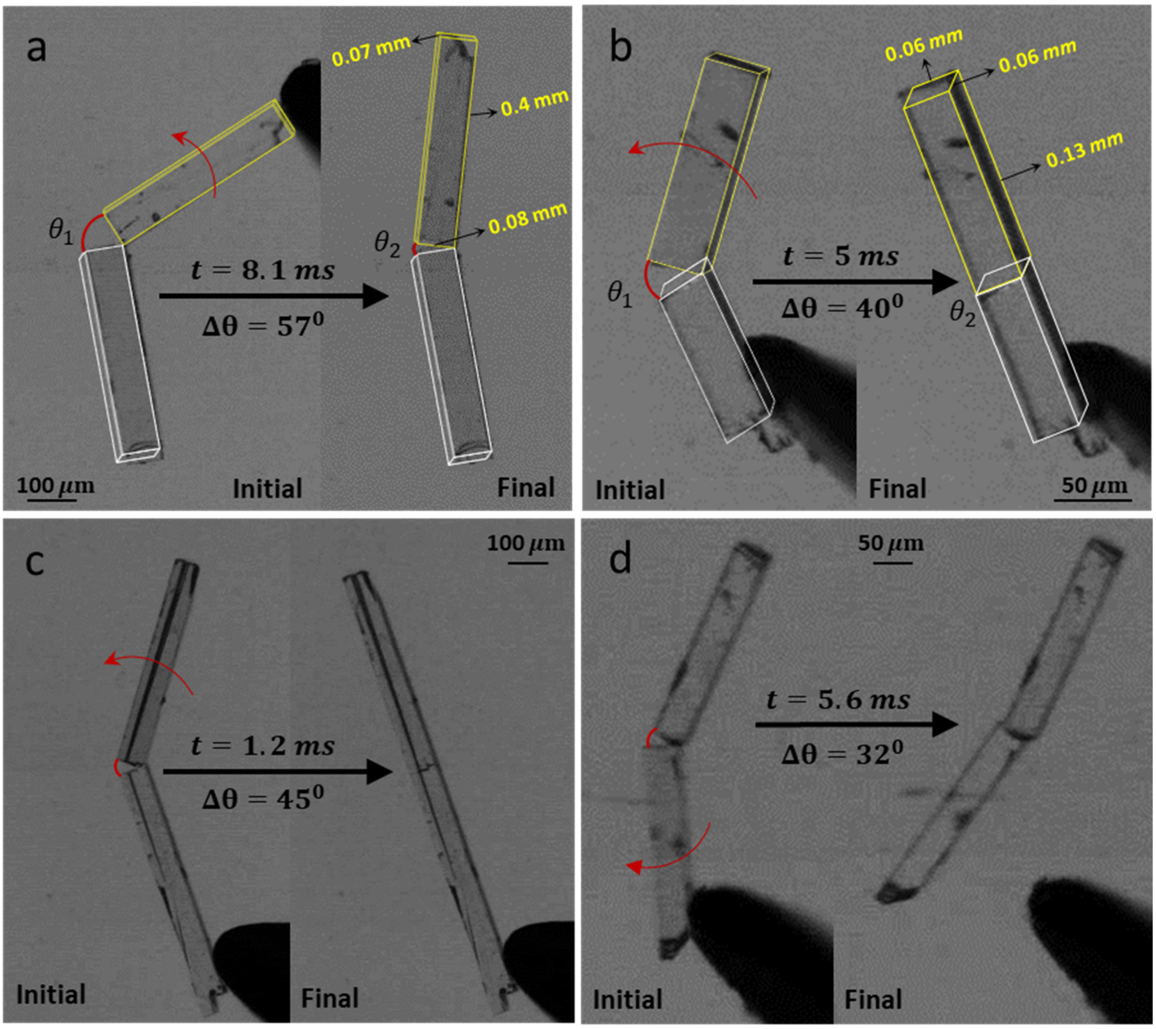

Supplementary Fig. 7: Calculation of actuation parameters using angular motion. a, **b**, **c**, **d** are the videograbs of four crystals (having specific dimensions of the actuating crystal part) during actuation following angular mechanics, where the actuation time is t; corresponding change of angle between initial and final frame during actuation is $\Delta\theta$, which were used to calculate the other parameters of actuation.

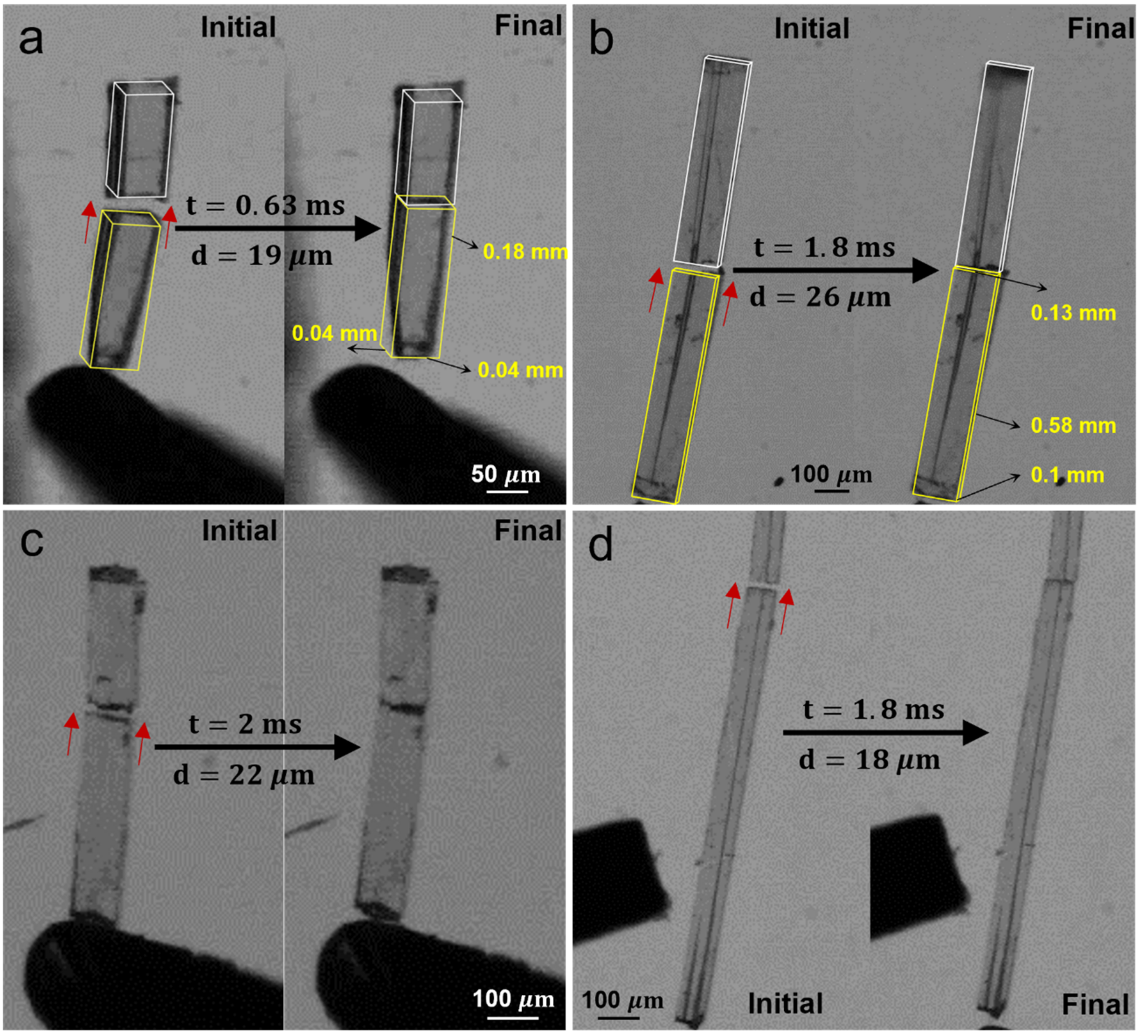

Supplementary Fig. 8: Calculation of actuation parameters using linear motion. a, **b**, **c**, **d** are the videograbs of four crystals (having specific dimensions of the actuating crystal part) during actuation following linear mechanics, where the actuation time is *t*; corresponding moving distance of actuating crystal part between initial and final frame during actuation is *d*, which were used to calculate the other parameters of actuation.

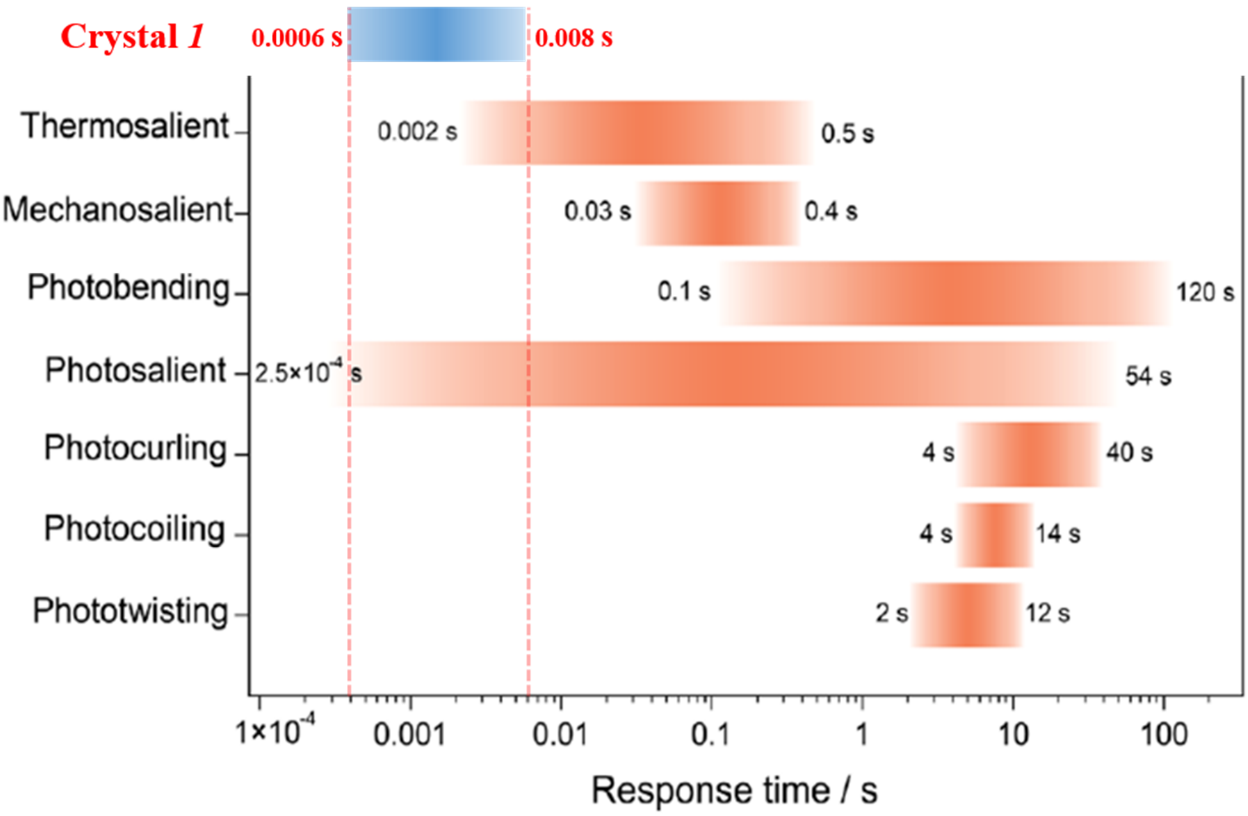

Supplementary Fig. 9: Response time of crystal ***1*** (**blue bar at the top**) with respect to the other reported stimuli responsive single crystals. Reproduced (and edited) with permission. Copyright Year 2020, American Chemical Society.[1]

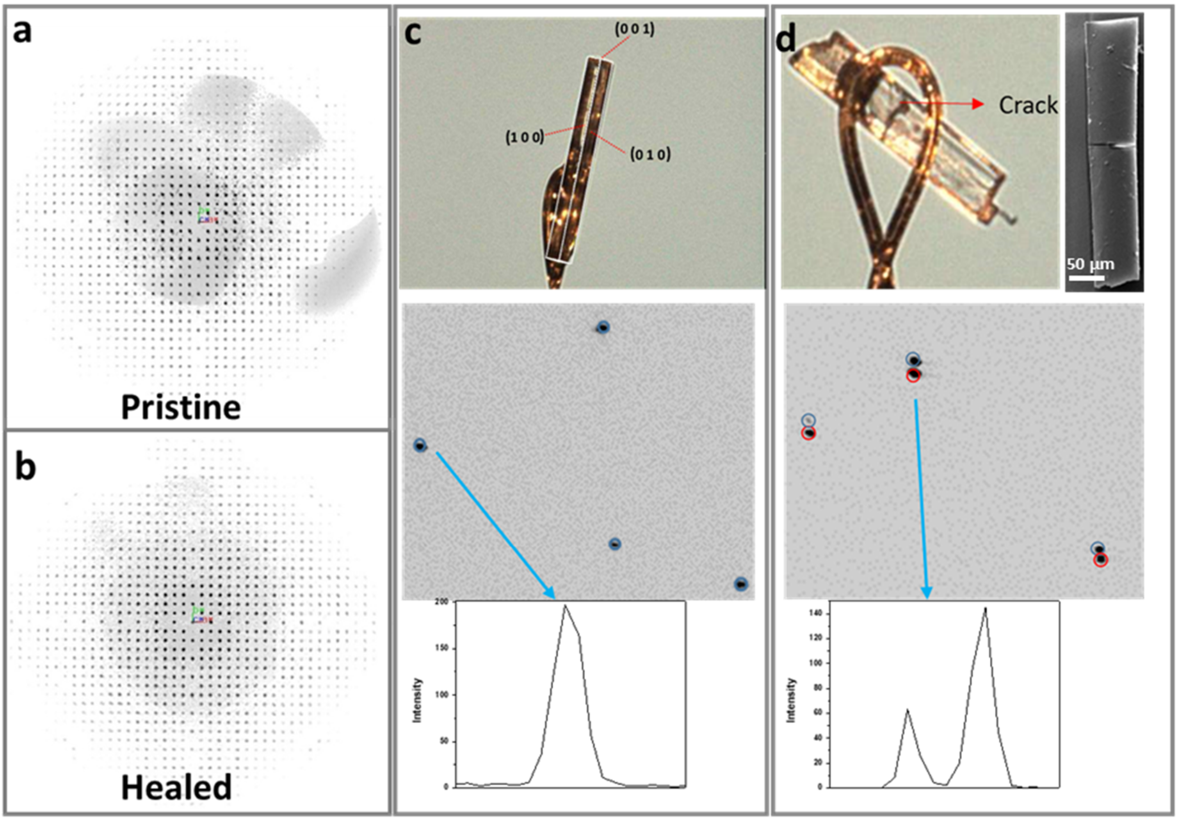

Supplementary Fig. 10: Examining self-healing efficiency using crystallographic diffraction experiments. a, **b** 2D projection of reconstructed 3D Ewald sphere of pristine and healed crystals, respectively, viewed along *c**; **c** optical image (top) showing face-indexed crystal (the length of the crystal ~ 800 μm), 2D diffraction patterns (bottom) and corresponding line profiles (intensity) of healed crystal (video-grab of healing event, seen in Supplementary Fig. 6b and Supplementary Movie 3); **d** crystal optical image (top-left) and SEM (top-right), 2D diffraction patterns (bottom) and corresponding line profiles (intensity) of a imperfectly healed crystal.

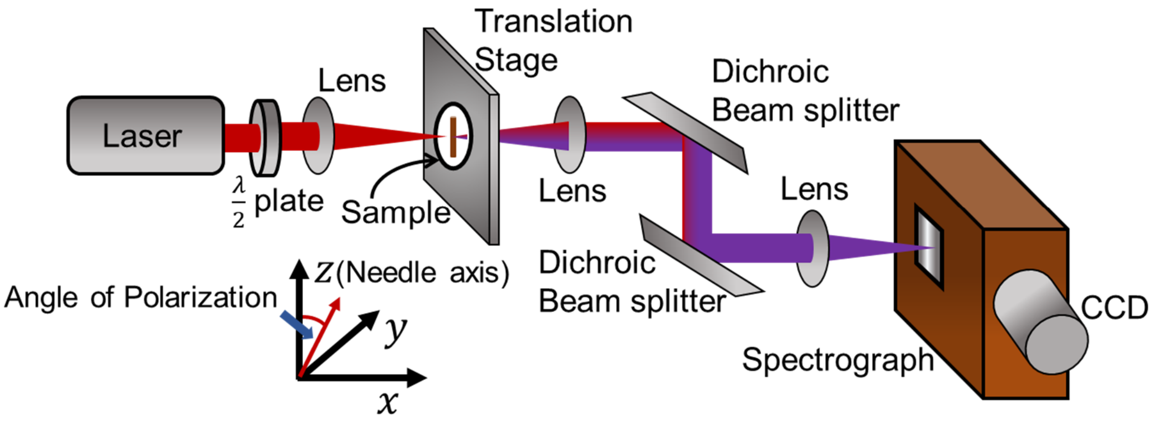

Supplementary Fig. 11: Schematic setup used for conducting SHG measurements.

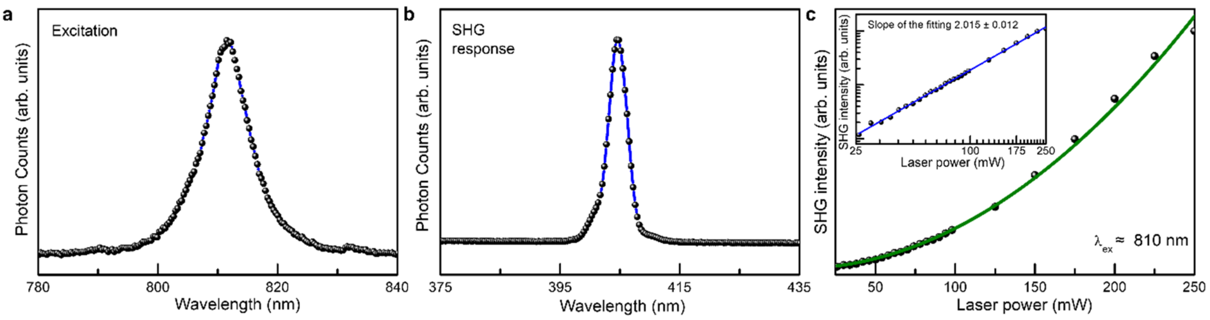

Supplementary Fig. 12: Characteristic spectra of SHG activity of crystal *1*. a, **b** intensity vs. wavelength spectra of incident beam and SHG signal, respectively; **c** intensity vs. excitation power in log-log scale.

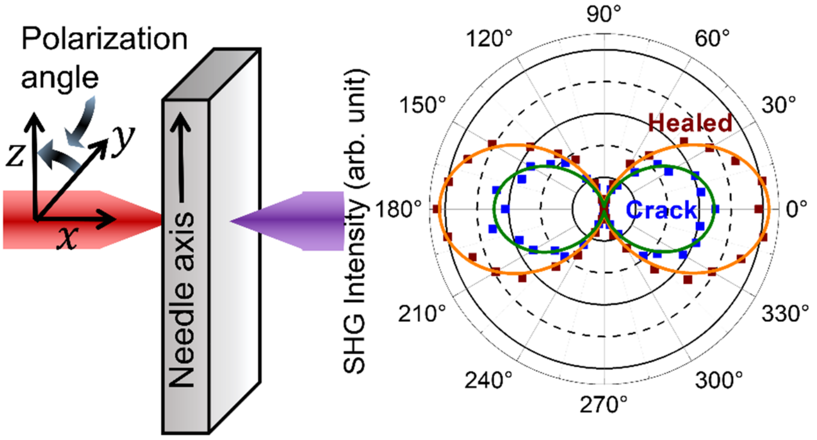

Supplementary Fig. 13: Polar plot of the SHG intensity from healed and imperfectly healed crystal (at crack junction) for different angle of linear polarization (angle of polarization has been measured with respect to the perpendicular, needle axis).

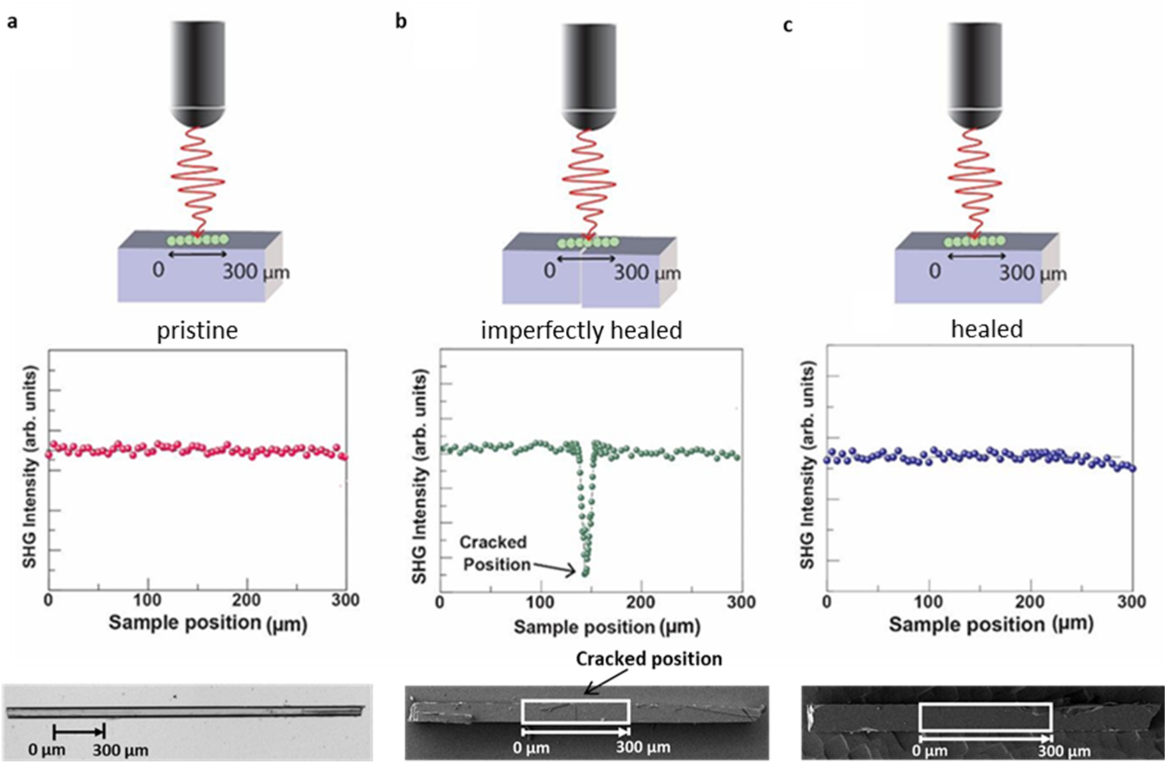

Supplementary Fig. 14: a, **b**, **c** *y*-polarized laser beam incident on (100) face of the pristine, imperfectly healed and healed crystals, respectively. A series of scanning SHG line-mapping measurements along *c*-axis of the crystals indicate that the SHG intensity suddenly drops at the vicinity of crack junction in case of imperfectly healed (see inset SEM image) crystal while in both the healed (see inset SEM image) and pristine (see inset optical image) crystals, the SHG intensity remains similar throughout the scan region.

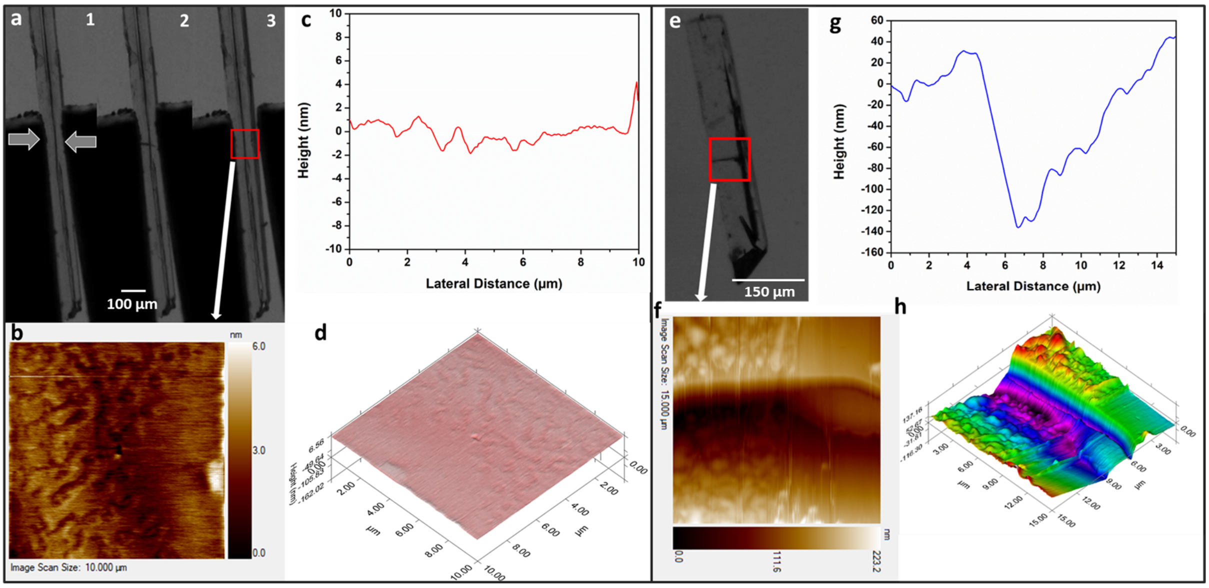

Supplementary Fig. 15: Videograbs of healed (see Supplementary Movie S5) and imperfectly healed crystals and their corresponding SPM images. a, **e** videograbs of healed and imperfectly healed crystals and their corresponding 2D SPM images **b**, **f**; 3D SPM images **d**, **h**; height profiles **c**, **g** respectively.

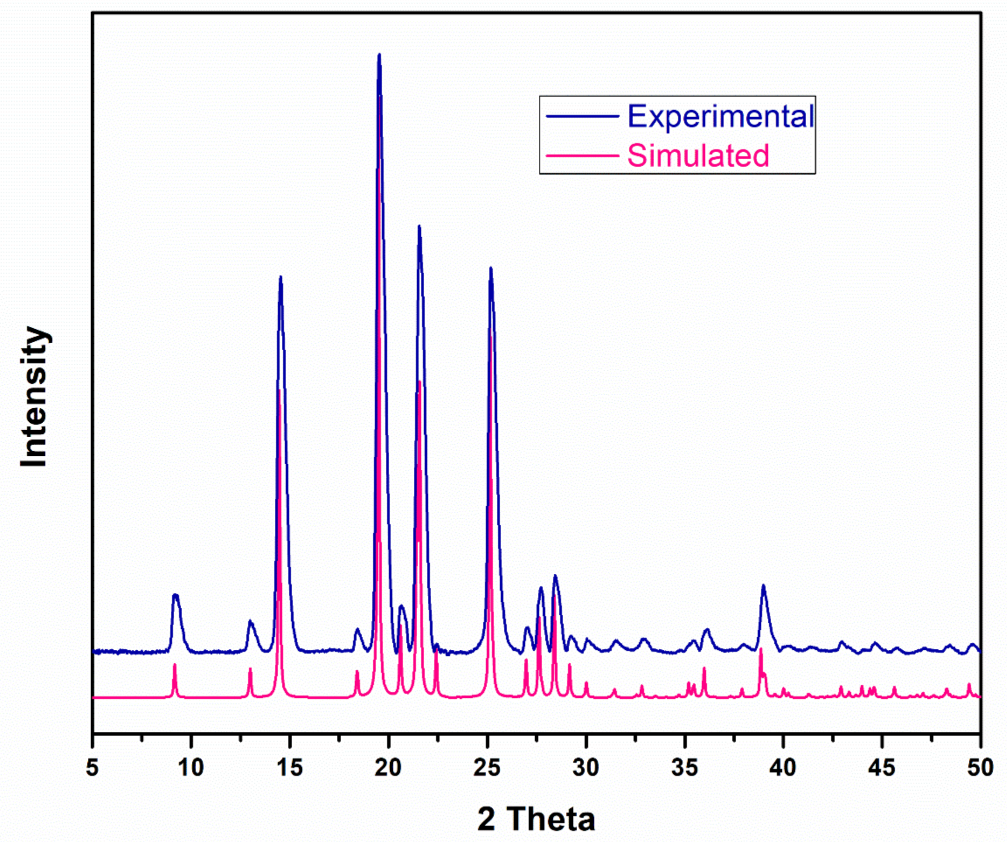

Supplementary Fig. 16: The phase purity of the bulk sample was confirmed by PXRD pattern of the simulated (pink) and experimental (blue) data.

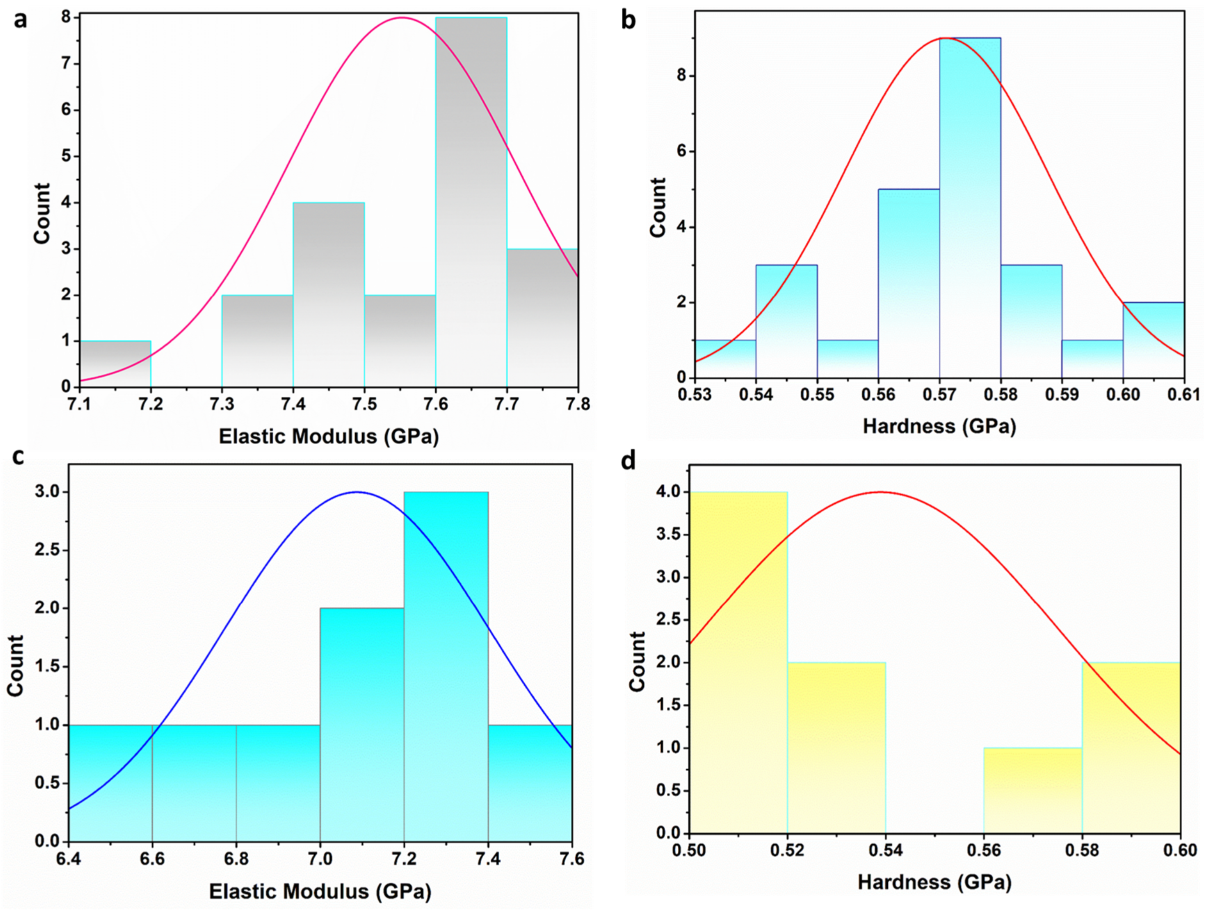

Supplementary Fig. 17: Histogram plots of elastic modulus for (100)/(010) face, (001) face **a**, **c** and hardness for (100)/(010) face, (001) face **b**, **d** of all indentations under 1 mN load obtained from several indentations from multiple crystals, respectively.

Supplementary Table 1. Quantified loads for multiple healing cycles and fracture for different crystals of ***1***.

Crystal Thickness	Healing cycles with applied load (mN)											Fracture load (mN)
	1st	2nd	3rd	4th	5th	6th	7th	8th	9th	10th	11th	
**80 *μ*m**	40	33	44	37	35	46	-	-	-	-	-	70
**86 *μ*m**	53	33	37	-	-	-	-	-	-	-	-	91
**83 *μ*m**	45	32	33	-	-	-	-	-	-	-	-	85
**92 *μ*m**	64	66	70	54	-	-	-	-	-	-	-	101
**79 *μ*m**	32	25	43	38	25	48	-	-	-	-	-	67
**92 *μ*m**	49	30	35	70	45	41	45	54	43	30	61	111
**96 *μ*m**	64	35	72	41	69	-	-	-	-	-	-	122
**93 *μ*m**	62	75	66	-	-	-	-	-	-	-	-	119
**61 *μ*m**	25	33	33	27	-	-	-	-	-	-	-	56
**88 *μ*m**	33	40	35	35	35	32	38	45	-	-	-	104
**98 *μ*m**	33	37	30	40	75	-	-	-	-	-	-	120
**72 *μ*m**	25	27	29	25	30	27	30	-	-	-	-	66

Supplementary Table 2. Ranges of materials properties and certain accessible performance attributes.

Angular Motion Performance Attributes		
**Attribute**	**Symbol [unit]**	**Corresponding Ranges**
Crystal dimensions	l x w x t [mm]	0.25 - 1.07, 0.04 - 0.12, 0.04 - 0.1
Moment of Inertia of actuating portion	I [Kgm^2]	4.3 x 10^{-18} – 8.9 x 10^{-16}
Actuation Time	t [ms]	0.62 - 8.13
Angular Acceleration	α [rads^{-2}]	13852 - 714002
Torque	τ [kgm^{2}s^{-2}]	0.15 x 10^{-12} - 96 x 10^{-12}
Work Capacity	W [Nm^{-2}]	0.18 – 15.2

Linear Motion Performance Attributes		
**Attribute**	**Symbol [unit]**	**Corresponding Ranges**
Crystal dimensions	l x w x t [mm]	0.3 - 3.0, 0.04 - 0.15, 0.04 - 0.15
Mass of the actuating portion	m [μg]	0.4 – 23.2
Actuation Time	t [ms]	0.6 - 5.00
Linear Acceleration	a [ms^{-2}]	1.05 - 62.4
Force Output	F [mN]	2.9 x 10^{-6} - 2.7 x 10^{-4}
Work Capacity	W [Nm^{-2}]	0.04 - 2

Supplementary Table 3. Crystallographic parameters of pristine and healed crystals.

Sample state	Pristine	Healed
Formula	**$C_{17}H_{18}N_2O_4$**	
Crystal System	**Tetragonal**	
Space group	***$I4_1cd$***	
a [Å]	19.25900(10)	19.25630(10)
c [Å]	8.69590(10)	8.69990(10)
V [Å ^{3}]	3225.39(5)	3225.97(5)
Z	8	8
Radiation	**CuK*α***	
ρ_{calcd} [gcm^{-3}]	1.295	1.294
F[000]	1328.0	1328.0
μ [mm^{-1}]	0.769	0.769
2θ [°]	9.184 to 136.394	9.186 to 136.434
hkl range	$-23 \le h \le 23$, $-23 \le k \le 23$, $-10 \le l \le 8$	$-23 \le h \le 23$ $-23 \le k \le 23$ $-10 \le l \le 9$
T [K]	293(2)	293(2)
reflns total	26827	27754
Criterion of observability	**$I > 2\sigma(I)$**	
Unique (obs/all)	1246/1320	1292/1344
R_1 (obs) / wR_2 (all)	0.0358/0.1014	0.0370/0.1100
GOF	1.051	1.101
CCDC number	2184474	2183527

Supplementary Table 4. Comparison of hydrogen bond geometries in pristine *vs* healed crystal.

D–H⋯A	d(D-H)/Å		d(H⋯A)/Å		d(D-A)/Å		D-H⋯A/°	
	**Pristine**	**Healed**	**Pristine**	**Healed**	**Pristine**	**Healed**	**Pristine**	**Healed**
N1–H1⋯O1	0.90(3)	0.94(3)	2.03(3)	2.00(3)	2.911(4)	2.910(4)	163.8(17)	163.5(16)
C3-H3⋯O1	0.93	0.93	2.49	2.49	3.261(4)	3.261(5)	140.00	140.00

[1]1-X,1-Y,+Z

Supplementary Table 5. Comparison of covalent bond distances in pristine *vs* healed crystal

**Atom groups**	**distance/Å**	
	**Pristine**	**Healed**
**O1–C8**	1.212(4)	1.212(4)
**O2–C8**	1.328(5)	1.326(5)
**O2–C9**	1.448(5)	1.444(6)
**N1–C2**	1.368(4)	1.370(4)
**N1–C1**	1.447(4)	1.449(4)
**C2–C7**	1.402(4	1.404(4)
**C2–C3**	1.399(4)	1.398(4)
**C8-C5**	1.470(5)	1.472(5)
**C5-C6**	1.384(5)	1.388(5)
**C5-C4**	1.399(4)	1.400(4)
**C7-C6**	1.376(5)	1.373(5)
**C3-C4**	1.367(4)	1.367(4)

Supplementary Table 6. Comparison of bond angles in pristine *vs* healed crystal.

Atom	Angle/°	
	**Pristine**	**Healed**
C8–O2–C9	115.8(3)	115.7(3)
C2-N1-C1	125.3(2)	125.5(2)
N1-C2-C7	123.8(3)	123.7(3)
N1-C2-C3	118.7(2)	118.8(2)
C3-C2-C7	117.5(3)	117.5(3)
O2-C8-C5	113.8(3)	114.0(3)
O1-C8-O2	122.9(4)	123.2(5)
O1-C8-C5	123.3(5)	122.8(5)
C6-C5-C8	120.8(3)	121.1(3)
C6-C5-C4	117.3(3)	117.0(3)
C4-C5-C8	121.9(3)	121.9(3)
C6-C7-C2	120.3(3)	120.3(3)
C4-C3-C2	121.4(2)	121.4(2)
N1-C1-N1^{1}	115.1(4)	115.1(4)
C7-C6-C5	122.3(3)	122.4(3)
C3-C4-C5	121.2(3)	121.4(3)

Supplementary Table 7. Calculations of angular actuation motion in single crystals of ***1*** (density = 1295kg/m^3).

SI. No	Cross-sectional area (mm^2)	Portion Volume (mm^3) x 10^{-2}	Actuating Mass (μg)	Actuation Time (ms)	Angular Displac-ement (rad)	Angular Acceleration (rad/s^2)	Moment of Inertia (kgm^2) x 10^{-16}	Torque (Kgm^2/sec^2) x 10^{-12}	Work Capacity (Nm^{-2})
1.	0.004	0.06	0.80	2.5	0.9	147103	0.081	1.20	1.78
2.	0.004	0.14	1.78	6.25	1.8	46203	0.71	3.27	4.28
3.	0.008	0.22	2.85	5.6	0.9	27194	0.94	2.55	1
4.	0.006	0.07	0.89	2.4	1	177179	0.06	1.02	1.51
5.	0.005	0.12	1.52	7	1.9	39067	0.33	1.3	2.11
6.	0.008	0.23	2.92	5	1	38546	0.92	3.56	1.52
7.	0.005	0.17	2.25	8	0.9	13852	0.96	1.33	0.68
8.	0.003	0.04	0.5	3.2	1.3	129647	0.04	0.55	1.91
9.	0.012	0.64	8.35	3.2	1.1	108162	7.91	85.54	14.7
10.	0.005	0.07	0.85	1.6	0.3	133658	0.07	0.92	0.48
11.	0.005	0.06	0.78	5	0.7	27643	0.06	0.15	0.18
12.	0.008	0.49	6.32	5.63	1.3	41084	8.91	36.62	9.75
13.	0.005	0.17	2.18	1.88	0.8	215920	0.82	17.7	8.00
14.	0.002	0.05	0.59	5.6	0.6	17676	0.12	0.21	0.25
15.	0.006	0.25	3.25	8.13	1.00	15140	1.97	2.98	1.19
16.	0.004	0.14	1.77	1.88	0.6	168262	0.66	11.11	4.80
17.	0.007	0.3	3.89	2.5	0.66	105210	2.76	29.00	6.35
18.	0.007	0.24	3.12	3.75	0.45	31648	1.49	4.72	0.87
19.	0.004	0.18	2.28	0.62	0.28	714002	1.34	96.00	15.21
20.	0.009	0.36	4.66	5	0.57	22962	2.56	5.88	0.94

Supplementary Table 8. Calculations of linear actuation motion in single crystals of ***1*** (density = 1295kg/m^3).

Sl. No	Cross-sectional area (mm^2)	Portion Volume (mm^3) x 10^{-2}	Actuating Mass (μg)	Actuation Time (ms)	Linear Displacement (μm)	Linear Acceleration (m/s^2)	Force (mN) x 10^{-4}	Work Capacity (Nm^{-2})
1.	0.023	1.07	13.9	2.4	87	15.12	2.10	1.71
2.	0.006	0.44	5.7	3.75	38	2.74	0.16	0.14
3.	0.007	0.24	3.08	1.88	17	4.86	0.15	0.11
4.	0.009	0.48	6.17	1.88	21	5.86	0.36	0.16
5.	0.021	1.5	19.25	4.38	35	1.82	0.35	0.08
6.	0.005	0.21	2.72	5	26	1.05	0.03	0.04
7.	0.001	0.06	0.8	1.25	22	13.88	0.11	0.4
8.	0.011	0.36	4.66	2.4	31	5.4	0.25	0.22
9.	0.011	1.8	23.25	1.88	26	7.52	1.75	0.26
10.	0.013	0.70	9.08	2.5	51	8.14	0.74	0.54
11.	0.01	0.22	2.85	3.13	48	4.9	0.14	0.3
12.	0.006	0.31	4.05	3.13	20	2.06	0.08	0.05
13.	0.01	0.47	6.14	4.38	47	2.46	0.15	0.15
14.	0.015	0.66	8.57	1.88	41	11.7	1.00	0.62
15.	0.012	0.93	12.1	3.13	27	2.73	0.33	0.1
16.	0.014	0.82	10.65	1.88	26	7.41	0.79	0.25
17.	0.008	0.20	2.53	1.88	17	4.81	0.12	0.11
18.	0.002	0.03	0.4	0.62	19	48.56	0.2	1.2
19.	0.018	1.02	13.26	1.25	32	20.25	2.7	0.83
20.	0.012	0.2	2.60	1.25	37	23.94	0.62	1.16
21.	0.005	0.12	1.61	1.88	22	6.2	0.1	0.17
22.	0.006	0.1	1.28	1.88	36	10.11	0.13	0.47
23.	0.004	0.11	1.46	0.62	24	62.4	0.9	1.97

Supplementary References:

1. Naumov, P. et al. The rise of the dynamic crystals. *J. Am. Chem. Soc.* **142**, 13256-13272 (2020).
2. Bhunia, S. et al. Autonomous self-repair in piezoelectric molecular crystals. *Science* **373**, 321-327 (2021).
