# Supplementary material for: Autonomous self-healing organic crystals for nonlinear optics
Source: Nat Commun. 2023 Oct 18;14:6589. doi: 10.1038/s41467-023-42131-7 (PMC10584936; doi:10.1038/s41467-023-42131-7)
Supplement: Supplementary file 3 — Description of Additional Supplementary Files [file 41467_2023_42131_MOESM3_ESM.pdf]

Description of Additional Supplementary Files

File Name: Supplementary Movie 1
Description: Autonomous self-healing of a mechanically fractured single crystal of 1 (sample 1) using a pair of needles (black object at rightside) and forceps (black object at left-side) shown in slow-motion (played at 30 fps) captured at 1250 fps under an optical stereo microscope.

File Name: Supplementary Movie 2
Description: Self-healing of a highly fractured single crystal of 1 (sample 2) by precise recombination, shown in slow-motion (played at 30 fps) and captured at 1600 fps. The video is mainly captured for healing time calculation.

File Name: Supplementary Movie 3
Description: The video of self-healing single crystal of 1 (sample 3) shown at slow-motion (played at 30 fps) captured at 1250 fps. The healed crystal of this video mainly used for SCXRD to check retention of bulk crystallinity.

File Name: Supplementary Movie 4
Description: Self-healing of a single crystal of 1 (sample 4) shown at slow-motion (played at 30 fps) captured at 1250 fps. The healed crystal mainly used to check SHG response at healed portion of the crystal.

File Name: Supplementary Movie 5
Description: Uniaxial force applied to fracture a single crystal of 1 (sample 5) by using a forceps (black object at top and bottom side of crystal) and upon release of the force the crystal self-heals autonomously, used for SPM imaging at healed region, shown in slow-motion (played at 30 fps) and captured at 1250 fps.

File Name: Supplementary Movie 6
Description: Repeatability of self-healing demonstrated in single crystals of 1 (sample 6). When the crystals were pushed gently against a force sensor, a visible crack generates and disappears upon withdrawal of the force and the process can be seen repeatable for several times. The movies are captured at real time.

File Name: Supplementary Movie 7
Description: Repeatability of self-healing demonstrated in single crystals of 1 (sample 7). When the crystals were pushed gently against a force sensor, a visible crack generates and disappears upon withdrawal of the force and the process can be seen repeatable for several times. The movies are captured at real time.

File Name: Supplementary Movie 8
Description: Uniaxial force applied to fracture a single crystal of 1 (sample 8) by using a forceps (black object at top and bottom side of crystal) and upon release of the force the crystal self-heals autonomously. The process repeatably continues for 10 cycles. The movie is captured at real time.

File Name: Supplementary Movie 9

Description: A single crystal of 1 (sample 9) is seen actuating angularly (analogous to a clock dial) in every frame until it reaches its static counter fragment. The video is recorded under 1600 fps and further used (along with other videos) for calculation of performance parameters.

File Name: Supplementary Movie 10
Description: Automated dynamics is seen in a mechanically altered single crystal of 1 (sample 10) that was recorded at 1600 frames per second and played back at 1 fps. As the actuating half re-joins the other part, the '>' shaped aperture is seen to close.

File Name: Supplementary Movie 11
Description: Two pieces of a mechanically separated (by three-point bending) single crystal of 1 (sample 11) self-actuate towards one another, which was captured at 1250 frames per second using a high-speed camera (shown at 1 fps). The shards are parallel to each other, attracting in a linear/straight fashion.

File Name: Supplementary Movie 12
Description: Ultrafast linear mechanical actuation of crystal 1 (sample 12) when broken gently into two pieces and placed opposite to each other. The video is recorded at 1600 fps and played at 1 fps. The lower crystal fragment can be seen actuating towards its counterpart.

File Name: Supplementary Movie 13
Description: Strong attraction forces draw one fragment of a single crystal of 1 (sample 13) toward its corresponding counterpart on its own, confirming the presence of strong columbic charges on the broken surfaces. The video is recorded at 1600 fps and played at 1fps.
